# Translation selectively destroys non-functional transcription complexes

**DOI:** 10.1038/s41586-023-07014-3

**Published:** 2024-02-07

**Authors:** Jason Woodgate, Hamed Mosaei, Pavel Brazda, Flint Stevenson-Jones, Nikolay Zenkin

**Affiliations:** Centre for Bacterial Cell Biology, Biosciences Institute, Faculty of Medical Sciences, Newcastle University, Newcastle Upon Tyne, UK

**Keywords:** DNA damage and repair, Transcription, Ribosome, Bacterial transcription, Genomic instability

## Abstract

Transcription elongation stalls at lesions in the DNA template^1^. For the DNA lesion to be repaired, the stalled transcription elongation complex (EC) has to be removed from the damaged site^2^. Here we show that translation, which is coupled to transcription in bacteria, actively dislodges stalled ECs from the damaged DNA template. By contrast, paused, but otherwise elongation-competent, ECs are not dislodged by the ribosome. Instead, they are helped back into processive elongation. We also show that the ribosome slows down when approaching paused, but not stalled, ECs. Our results indicate that coupled ribosomes functionally and kinetically discriminate between paused ECs and stalled ECs, ensuring the selective destruction of only the latter. This functional discrimination is controlled by the RNA polymerase’s catalytic domain, the Trigger Loop. We show that the transcription-coupled DNA repair helicase UvrD, proposed to cause backtracking of stalled ECs^3^, does not interfere with ribosome-mediated dislodging. By contrast, the transcription-coupled DNA repair translocase Mfd^4^ acts synergistically with translation, and dislodges stalled ECs that were not destroyed by the ribosome. We also show that a coupled ribosome efficiently destroys misincorporated ECs that can cause conflicts with replication^5^. We propose that coupling to translation is an ancient and one of the main mechanisms of clearing non-functional ECs from the genome.

## Abstract (web summary)

Translation actively dislodges stalled transcription elongation complexes (ECs) from damaged DNA, which enables lesion repair and restoration of transcription activity, and coupled ribosomes discriminate between active ECs and stalled ECs, ensuring destruction of only the latter.

## Main

**Fig. 1:**
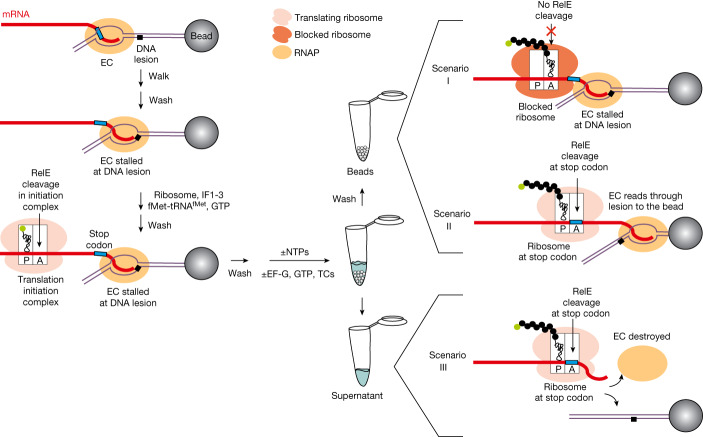
Experimental setup for analysis of encounters between a stalled EC and coupled translating ribosome. The left side illustrates the assembly of the coupled transcription–translation system immobilized on streptavidin beads (for sequences, see Extended Data Fig. 1). On the right are the possible outcomes of the ribosome catching up with the stalled EC. The cartoon shows where (beads or supernatant) components of the system can be found after the encounter of translating ribosome with stalled EC, and where cleavage by RelE is expected in each scenario.

**Fig. 2:**
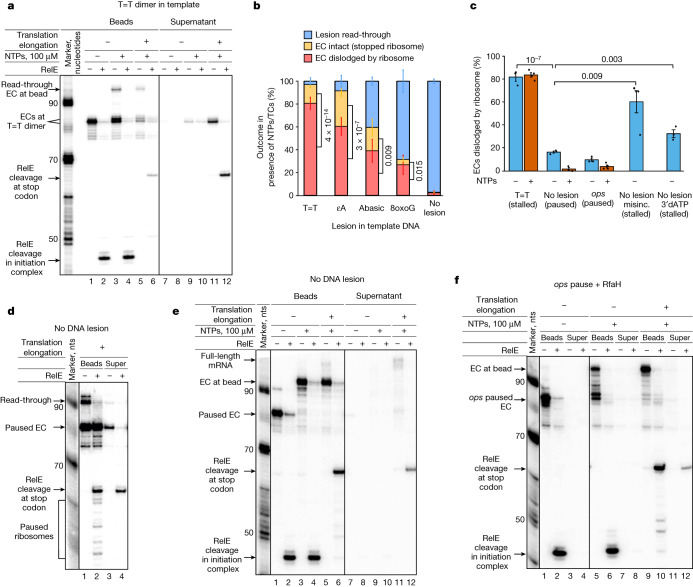
Coupled translating ribosome dislodges stalled ECs but not paused ECs. **a**, Dislodging of ECs stalled at the T=T lesion by a translating ribosome. Note that RelE cleavage at the stop codon of mRNA that remained on the beads takes place in the coupled ECs that read through the lesion (see Extended Data Fig. 2a for an explanation of the pattern). **b**, EC fate on different DNA lesions during coupled transcription–translation (also Extended Data Fig. 3). ECs that read through the lesion are blue; ECs that were dislodged by ribosome are pink; ECs that were not dislodged and caused the stop of the ribosome are yellow. Data are presented as mean values ± s.d. from three to four biological replicates; *P* values (two-sided Student’s *t*-test) for dislodged versus non-dislodged ECs are shown next to the bars. **c**, Functional (in the absence of NTPs; blue) and kinetic (in the presence of NTPs; red) discrimination between stalled and paused ECs during dislodging by a coupled ribosome (also **d**–**f** and Extended Data Fig. 3e,f). Data are presented as mean values ± s.d. from three or more biological replicates (dots); *P* values (two-sided Student’s *t*-test) are shown above the histogram. **d**, Coupled translation has little effect on ‘active’ ECs paused by NTP deprivation. Note that RelE cleavage at the stop codon in the complexes that remained on the beads takes place from coupled ECs that read through the pause site during the initial walking of EC (see explanation of the cleavage pattern in Extended Data Fig. 2b). Note also that some ribosomes pause before reaching the EC (RelE cleavage at earlier codons). **e**, Simultaneous transcription and translation elongation does not lead to dislodging of active ECs. **f**, A coupled ribosome does not dislodge ECs paused at the *ops* pause sequence but rather helps them into elongation. **a**,**d**–**f**, All experiments were repeated independently three or more times with similar results. For gel source data, see Supplementary Fig. 1.

**Extended Data Fig. 1:**
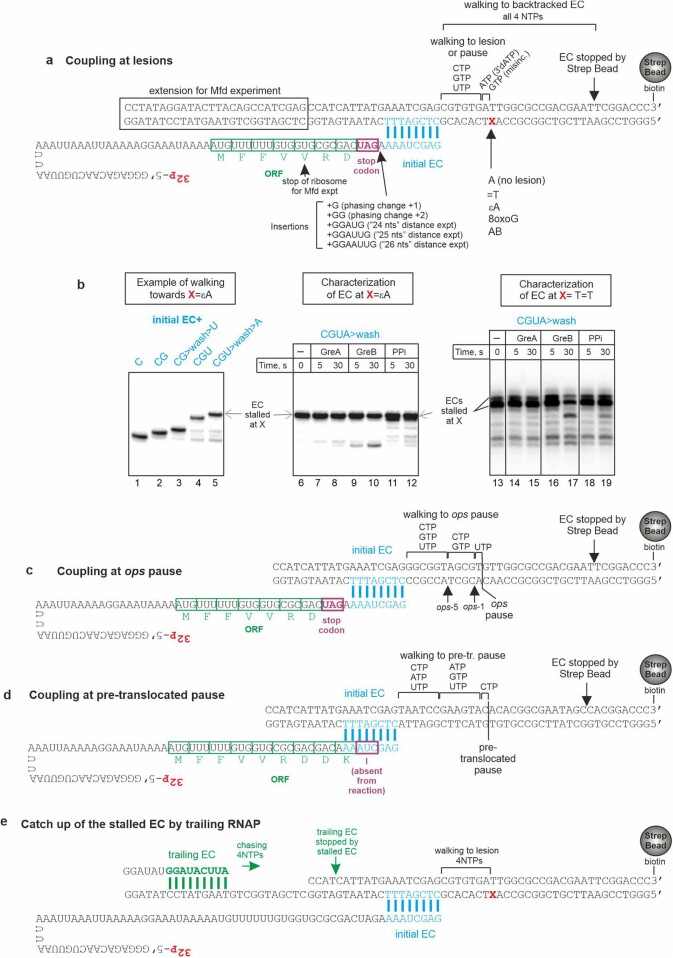
Nucleic acids of in vitro coupled transcription-translation system. **a**, **c**–**e**. Nucleic acids of assembled ECs, and steps of their walking to positions of stalls and/or pauses. Ribosome was allowed to translate to the stop codon or to the marked position. DNA extension used in Mfd experiments is framed in panel a. **b**. Example of walking of initial EC to the site of DNA lesion, and characterization of the ECs stalled at T = T and εA lesions using GreA, GreB and pyrophosphate cleavage. Note that, at T = T lesion, the stalled EC undergoes slow incorporation of AMP, with the resulting EC being also in the stalled state. For gel source data see Supplementary Fig. 1. Experiments were repeated independently at least 3 times with similar results.

RNA polymerase (RNAP) may pause on encountering lesions in the DNA template that result from ionizing radiation, ultraviolet (UV) irradiation, and chemical and enzymatic reactions^6^. These DNA lesions include pyrimidine dimers (such as thymine dimers, T=T), 8-oxoguanine (8oxoG), abasic sites and 1,*N*^6^-ethenoadenine (*ε*A). We recently showed that a coupled translating ribosome can rescue transcription elongation complexes (ECs) from backtracking by physically pushing them forward^7^. We asked how EC stalling at DNA lesions would be affected by coupled translation. To investigate this, we used an in vitro coupled transcription–translation system assembled from purified components^7,8^ (Fig. 1). The EC was immobilized on streptavidin beads by a biotin linker on the non-template DNA strand, challenged with high ionic strength to remove misfolded ECs and walked to the lesion in the template strand (Fig. 1 and Extended Data Fig. 1a,b). A translation initiation complex was then formed on the messenger RNA (mRNA) of the EC using purified ribosomes, initiation factors and initiating fMet-tRNA^fMet^. Translation was then started by addition of individual ternary complexes (complexes of aminoacylated transfer RNAs (tRNAs) with EF-Tu/GTP (guanosine triphosphate)) and EF-G/GTP. The mRNA contained a stop codon 19 nucleotides (nts) from the 3′ end of mRNA. As the minimal possible distance between active centres of a coupled EC and ribosome is 25–26 nts (ref. ^7^), this stop codon can be reached by the translating ribosome only if the stalled EC has vacated the lesion site. Immobilization on the beads allowed washing of the coupled system at any step, and identification of any components that have dissociated from the coupled system (Fig. 1). The position of the ribosome on mRNA and the efficiency of coupling with EC was detected by specific cleavage of mRNA in the vacant A-site of the ribosome by the toxin RelE^7^ (for example, Fig. 2a, lanes 1 and 2). Three outcomes that can be expected on simultaneous addition of nucleoside triphosphates (NTPs) and the start of translation elongation are shown in Fig. 1.

**Extended Data Fig. 2:**
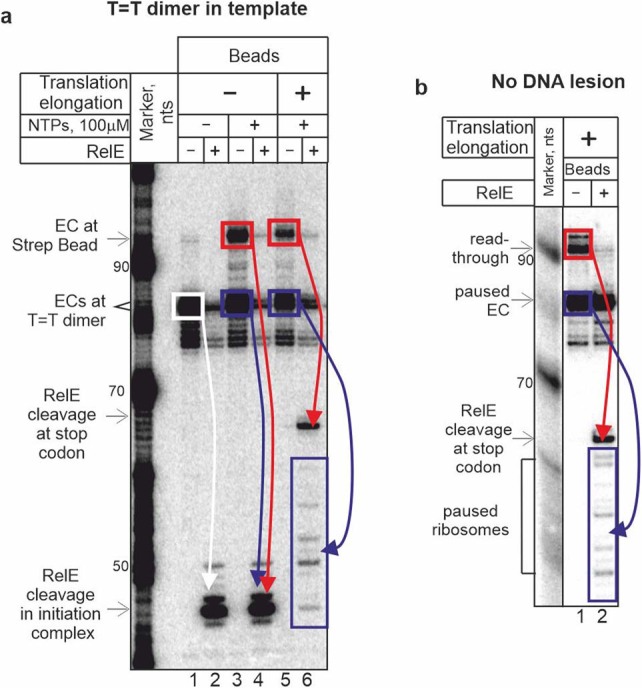
Pattern of RelE cleavage in the coupled system (on the example of Fig. 2a,d). **a**. RelE cleavage at the stage of translation initiation takes place in both stalled (or paused) ECs and ECs that read-through the lesion (or pause) (lanes 1–4). **a**-**b**. After translation elongation, RelE cleavage at stop codon proceeds only from read-through ECs (ribosome that collided with EC stops with A-site occupied by peptidyl-tRNA and cannot be cleaved by RelE) (a, lanes 5-6, and b, lanes 1-2). Some of RelE cleavage in stalled/paused ECs that were not dislodged by ribosome takes place at distant codons, indicating that ribosome slows down through pausing when approaching a “stable” EC (a, lane 6, and b, lane 2).

**Extended Data Fig. 3:**
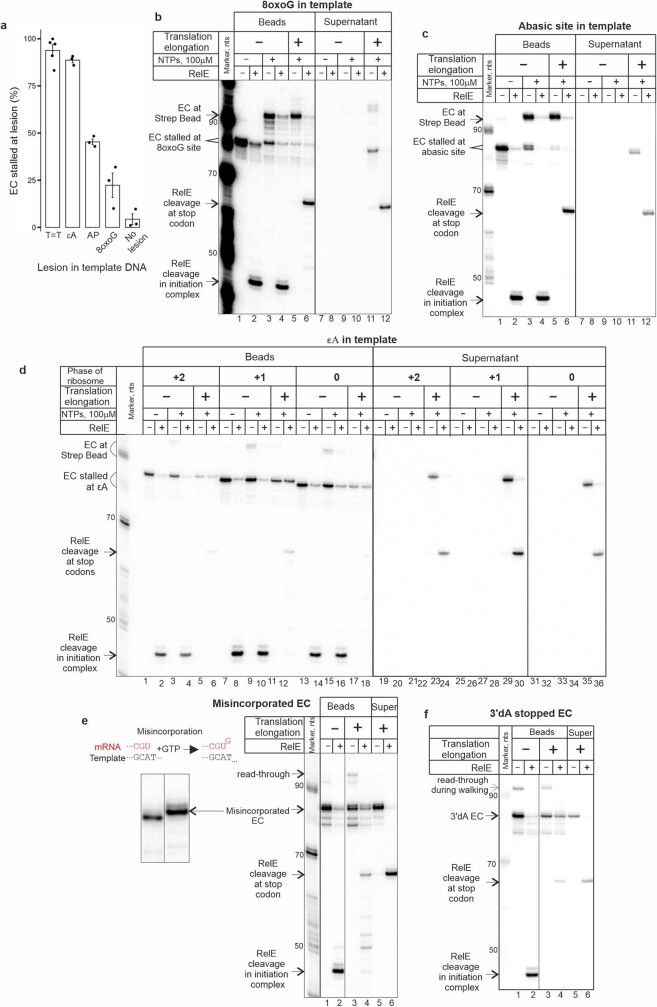
Translating ribosome dislodges stalled ECs. **a**. Stalling of EC at different lesions (without translation elongation; compare to Fig. 2b). Data are presented as mean values ± SD from ≥3 biological replicates (dots); *p*-value (two-sided Student’s *t*-test) are shown above the histogram. **b**–**f**. Representative experiments of ribosome-mediated dislodging of ECs stalled at various lesions or after incorporating wrong substrates. Note, that, in the presence of NTPs, some of the ECs stalled at 8oxoG and AP lesions undergone one nucleotide incorporation but remained stalled. Note also that RelE cleavage at the stop codon of mRNA that remained on the beads takes place in the coupled ECs that read-through the lesion. For gel source data see Supplementary Fig. 1. Experiments were repeated independently at least 3 times with similar results, and quantitative analysis is shown in Fig. 2b,c.

As can be seen from Fig. 2a, in the presence of NTPs alone, only a small portion of the ECs reads through the T=T lesion (lane 3; note that read-through ECs remained in ‘beads’ fraction being stopped by streptavidin at the end of DNA duplex) with most ECs remaining stalled at the lesion site. In the presence of NTPs, some stalled ECs underwent slow incorporation of one nucleotide but remained stalled (two bands of the stalled ECs in Fig. 2a, lane 3; the same was observed for 8oxoG and abasic sites, but not εA, Extended Data Figs. 1b and 3b–d). However, on addition of ternary complexes with EF-G/GTP, the coupled translating ribosome did not assist the EC in the read through of the lesion (Fig. 2a, compare lanes 3 and 5). Instead, translation led to the release of most stalled ECs into the supernatant (Fig. 2a, compare lanes 5 and 11). The released mRNA remained bound by the ribosome that, in the absence of an obstacle, had reached the stop codon (as judged by RelE cleavage; Fig. 2a, lane 12), suggesting that the translating ribosome was responsible for the destruction of the stalled ECs (scenario III in Fig. 1). ECs that remained stalled at the lesion in the beads fraction blocked translocation of the ribosome thus preventing RelE cleavage (Fig. 2a lane 6; scenario I in Fig. 1) or were not even reached by the translating ribosomes as judged by RelE cleavage at distant codons (Extended Data Fig. 2a, lane 6). Note that RelE cleavage at stop codon in the beads fraction originated from the ECs that had read through the lesion (Fig. 2a, lane 6; Extended Data Fig. 2a, lane 6), because in such coupled complexes, the ribosome was able to reach the stop codon without dislodging the EC (scenario II in Fig. 1).

We analysed ECs stalled at some other DNA lesions (8oxoG, abasic sites and *ε*A). As expected^9^, the efficiency of read through varied for different lesions (Extended Data Fig. 3a). However, roughly 65–85% of ECs that remained stalled at the lesions were dislodged from the DNA by the translating ribosome (Fig. 2b). The different sensitivities of ECs, stalled at different lesions, to dislodging by the ribosome suggests that there are either functional variations among these ECs or different distributions of ‘stable’ versus ‘dismissible’ states at these DNA lesions.

## Ribosomes do not destroy active ECs

**Extended Data Fig. 4:**
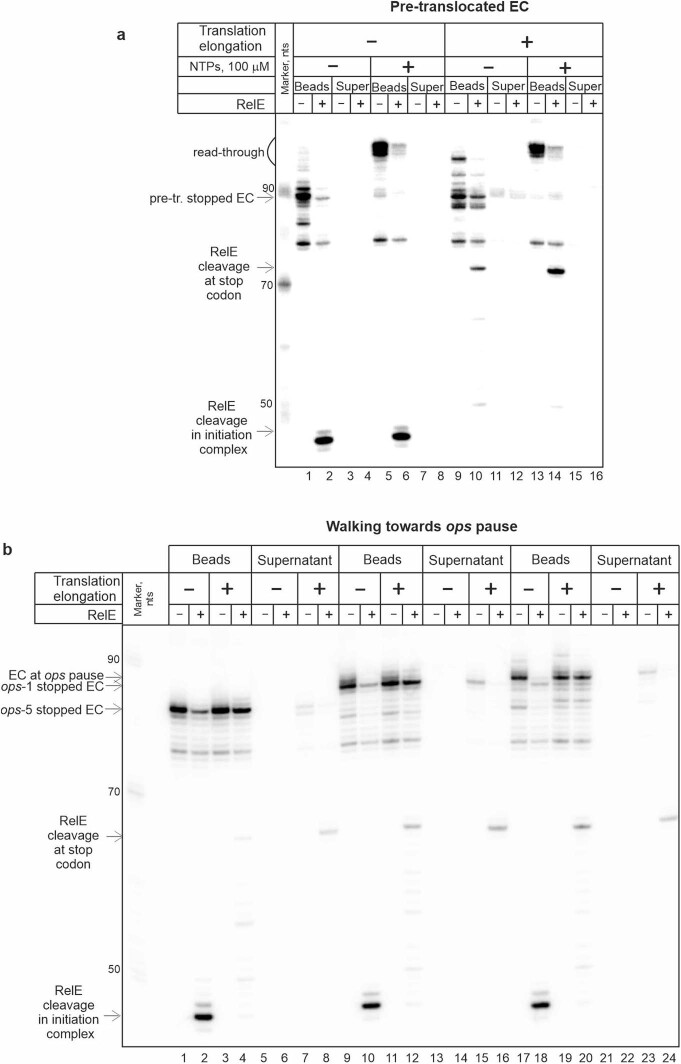
Coupled translating ribosome does not dislodge paused ECs. **a**,**b**. The walking steps for formation of “pre-translocated”, “ops-5”, “ops-1” and “*ops* pause” ECs are shown in Extended Data Fig. 1c,d. For gel source data see Supplementary Fig. 1. Experiments were repeated independently 3 times with similar results.

‘Functional’ ECs, paused on an undamaged DNA template in the absence of NTPs, were much less susceptible to destruction by the translating ribosome (Fig. 2c,d, compare lanes 1 and 3). Instead, translating ribosomes were blocked in the pretranslocated state by the paused EC, as judged by the absence of RelE cleavage (scenario I in Fig. 1), or could not even reach the paused EC, as judged by the cleavage at distant codons (Fig. 2d, lane 2). This suggests that there is a functional difference between a paused EC and an EC stalled at a DNA lesion that makes the latter susceptible to approach and to dismissal by the ribosome. Furthermore, in the presence of NTPs, almost all paused ECs extended mRNA without being affected by the translating ribosome (Fig. 2c,e, compare lanes 5 and 11). Such a kinetic checkpoint may further ensure that only stalled ECs, but not paused ECs, are dislodged by the coupled ribosome. Similar results (5–15% of ECs dislodged in the absence of NTPs) were observed with other paused ECs (Extended Data Fig. 4b, lanes 3, 7 and 11, 15), including an EC stabilized in the pretranslocated state by a specific RNA–DNA hybrid sequence^10^ (Extended Data Figs. 1d and 4a, lanes 9, 11).

Next, we tested ECs paused at the strong and well-characterized *ops* pause^11^ (Extended Data Fig. 1c). In the absence of NTPs, similarly to the paused ECs above, progression of most of the ribosomes was blocked by the ECs at the *ops* site with only few ECs being dislodged (Fig. 2c and Extended Data Fig. 4b, lanes 19, 23). As expected, in the presence of only NTPs, strong pauses of transcription formed (Fig. 2f, lane 5). However, the translating ribosomes did not dislodge those paused ECs either but, instead, ‘helped’ them to resume elongation (Fig. 2f, compare lanes 9 and 11). The result indicates that the ribosome is not only able to distinguish between paused and stalled ECs, dislodging only the latter, but also to assist the naturally paused ECs into elongation. Active assistance to backtracked and paused ECs by the ribosome was also reported earlier by us and others^7,12^.

## Dislodging of ECs is ribosome specific

**Extended Data Fig. 5:**
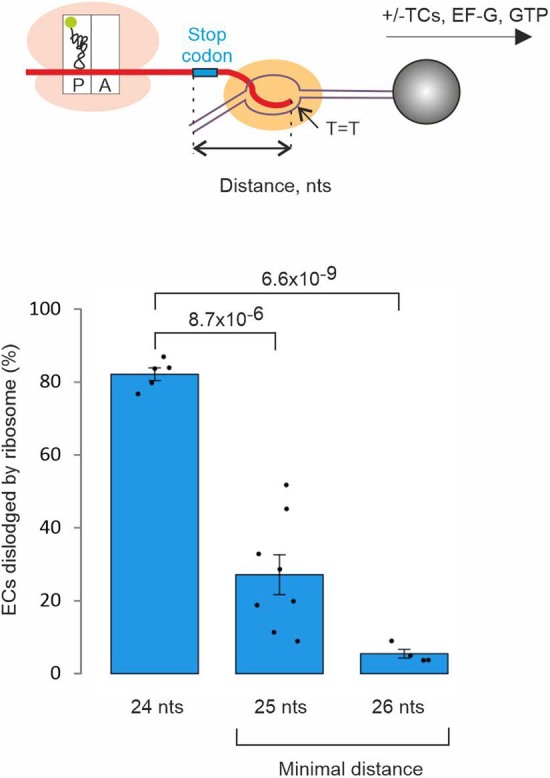
Dislodging of stalled EC by ribosome requires collision between machineries. Efficiency of EC dislodging by coupled ribosome strictly depends on the distance between machineries on mRNA. The distance was changed by introducing short sequences in mRNA between stop codon and the stalled EC (“24”, “25” and “26” templates in Extended Data Fig. 1a). Ribosome was allowed to translate to the stop codon, and outcome on the stalled EC was monitored. 25-26 nts between active centers of ribosome and RNAP is the minimal possible distance upon collision of two machineries. Data are presented as mean values ± SD from ≥3 biological replicates (dots); *p*-value (two-sided Student’s *t*-test) are shown above the histogram.

Stopping the ribosome on mRNA at distances larger than the minimal distance between coupled ribosome and EC (that is, 25–26 nts on mRNA between their active centres^7^) had no or little effect on the EC stalled at a lesion (Extended Data Fig. 5). This suggests that direct physical force applied by the translating ribosome on the stalled EC is required for EC destruction. A translating ribosome translocates along mRNA by steps of three nucleotides (codons), meaning that it can approach a stalled EC in three different phases, which may influence the outcome of their encounter. However, changing the phasing of the interaction by introducing one or two nucleotides in mRNA between the interacting machines did not affect the efficiency of dislodging of the stalled EC (Extended Data Fig. 3d).

**Extended Data Fig. 6:**
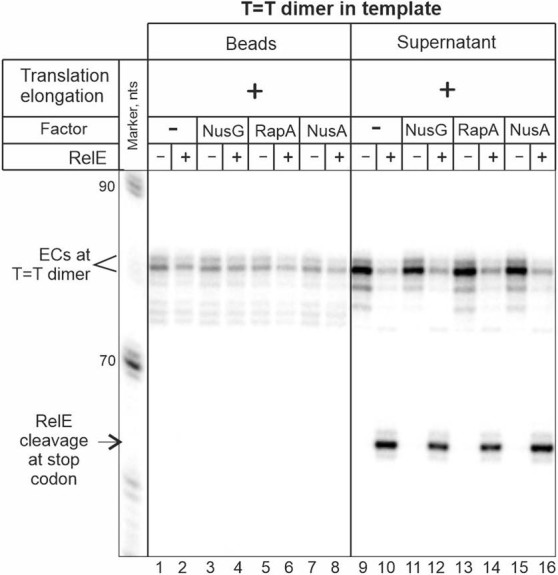
Effects of transcription factors on stalled EC dislodging. Coupling factors NusA and NusG and translocase RapA that binds at the ribosome-RNAP contact interface do not affect dislodging of a stalled EC. For gel source data see Supplementary Fig. 1. Experiments were repeated independently 3 times with similar results.

The transcription factors NusA and NusG are implicated in the coupling of transcription and translation^13–15^, whereas another factor, RapA, binds at the interface of interaction of the EC with a coupled ribosome^16^. Either of these factors may affect the outcome of interactions of the ribosome with an EC stalled at a lesion. We, however, found that neither of them affected the dislodging of the stalled EC by a coupled translating ribosome (Extended Data Fig. 6).

**Fig. 3:**
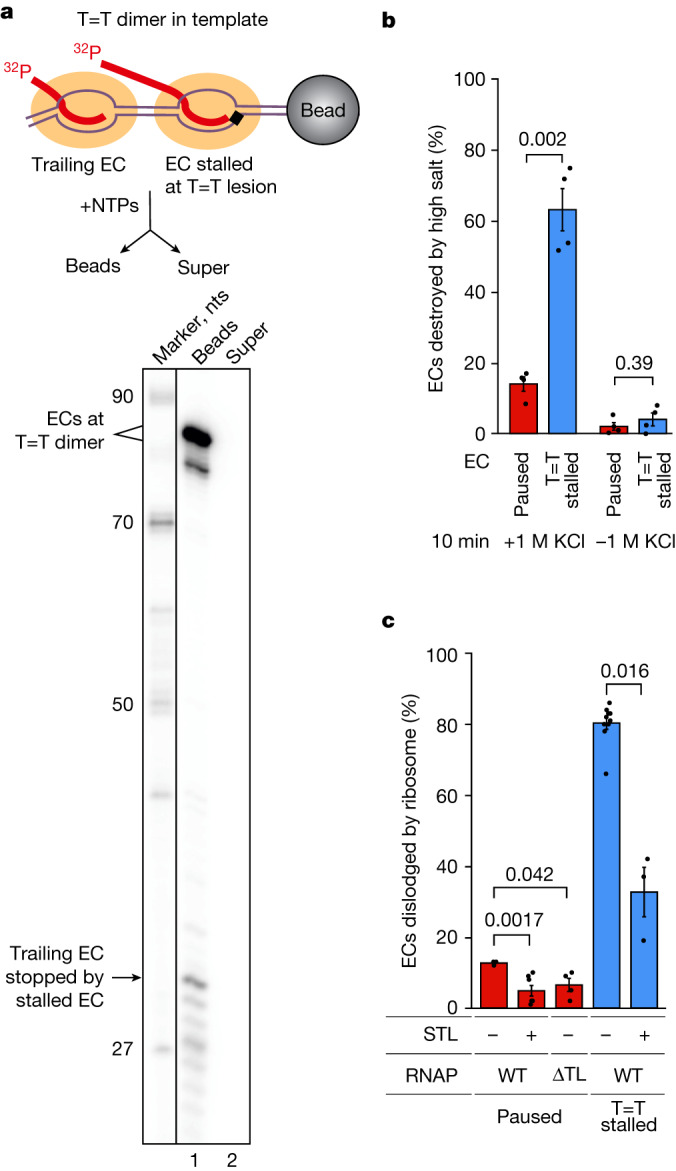
Stalled ECs are specifically recognized by the ribosome. **a**, A trailing EC does not dislodge EC stalled at the T=T lesion. The scheme of the experiment is shown above the gel. For gel source data, see Supplementary Fig. 1. The experiment was repeated independently three times with similar results. **b**, Salt stability of stalled and paused ECs. **c**, The Trigger Loop (TL) participates in determining the susceptibility of EC to ribosome-mediated destruction. **b**,**c**, Data are presented as mean values ± s.d. from three or more biological replicates (dots); *P* values (two-sided Student’s *t*-test) are shown above the histogram.

Dislodging of stalled ECs may not be specific to the ribosome. Therefore, we tested whether a stalled EC can also be dislodged by a trailing RNAP transcribing the same DNA: another frequent encounter expected for a stalled EC. The trailing EC was assembled behind the stalled EC (schemes in Fig. 3a and Extended Data Fig. 1e) and the system was supplied with NTPs. As can be seen from Fig. 3a, elongation by the trailing EC was blocked by the stalled EC, with the latter remaining intact, suggesting that dismissal of a stalled EC is ribosome specific.

## Structure of an EC stalled at the T=T lesion

**Extended Data Fig. 7:**
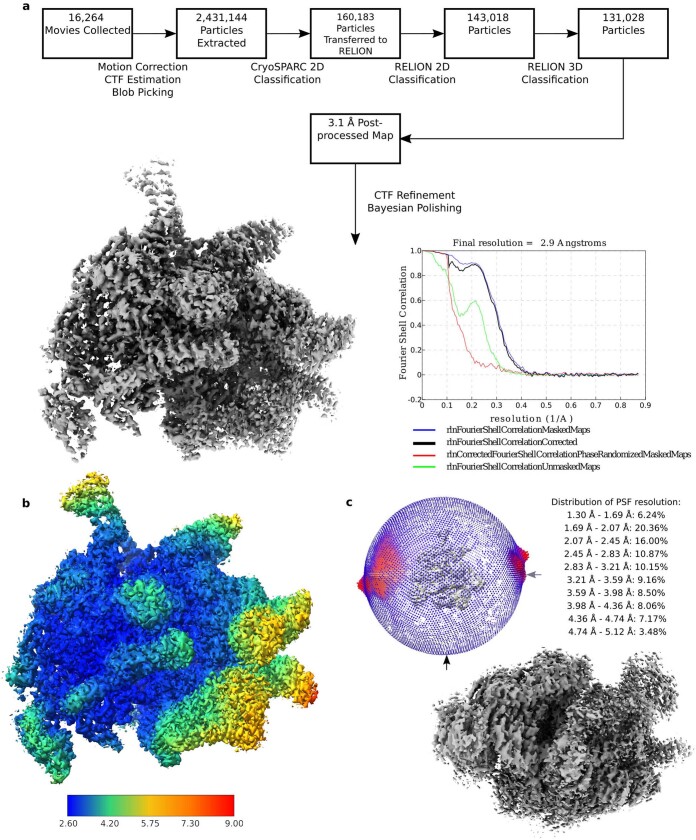
Cryo-EM Processing Pipeline. **a**. Workflow for generating final density map, followed by image of the final RELION auto refined and post-processed density map. To the right, post processed resolution statistics generated from RELION are shown. **b**. Final 3D auto refined density map with local resolution data (measured in Å). **c**. Angular distribution for particle projections, and view of the map (black arrow) perpendicular to the best orientation plane (grey arrow) to highlight the orientation bias causing smearing of the map. The view shown in panel (a) corresponds to the grey arrow. Table shows percentages of point spread function (PSF) resolution.

**Extended Data Fig. 8:**
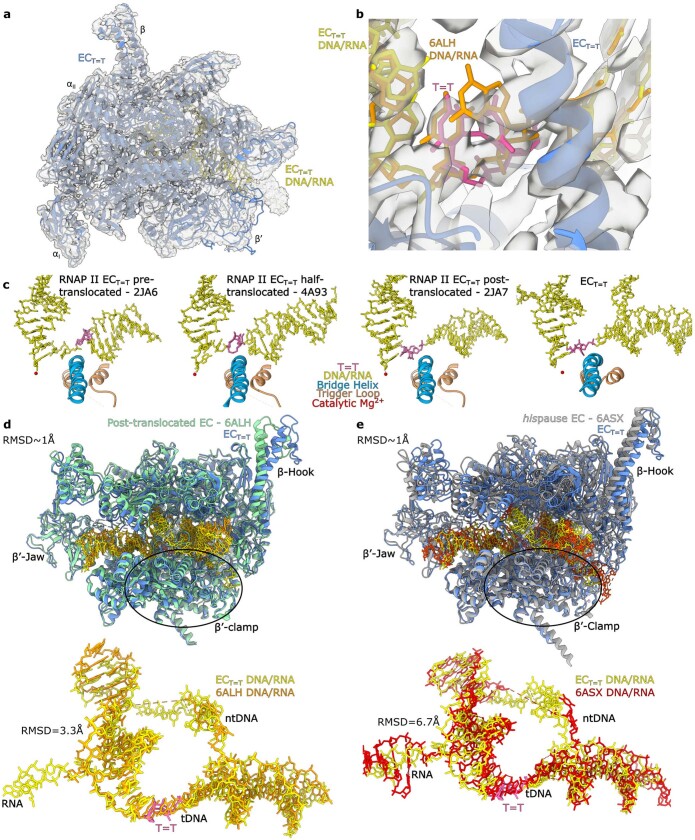
Modelling EC_T=T_. **a**. Overall fit of EC_T=T_ model to the 2.9 Å post-processed cryo-EM density map. **b**. Comparison of fitting into EC_T=T_ cryo-EM map of EC_T=T_ model (blue protein, yellow nucleic acids with pink T = T dimer) and canonical post-translocated EC (orange nucleic acids; PDB ID: 6ALH^17^), showing the overall better fit for the T = T dimer over the template nucleoside monophosphate of the post-translocated EC. **c**, Active centre of EC_T=T_ is similar to active centre of RNAP II after incorporation of AMP at the T = T lesion. Nucleic acids are yellow with magenta T = T dimer; catalytic Mg^2+^ is a red sphere; Bridge Helix (blue) and Trigger Loop (tan) are shown as cartoon. From the left: pre-translocated RNAP II EC_T=T_, when RNAP II encounters T = T lesion (PDB ID: 2JA6); half-translocated RNAP II EC_T=T_ overcoming the first translocation barrier (PDB ID: 4A93); post-translocated RNAP II EC_T=T_ after AMP incorporation (PDB ID: 2JA7); *E. coli* EC_T=T_. **d**, Comparison of EC_T=T_ model to canonical post-translocated EC (PDB ID: 6ALH^17^) with nucleic acid overlay comparison underneath. The protein is shown as cartoon with EC_T=T_ in blue and 6ALH in green. The nucleic acids are shown as sticks; EC_T=T_ in yellow with pink T=T, and 6ALH in orange. **e**, Comparison of EC_T=T_ model to half-translocated *his*pause EC (PDB ID: 6ASX^18^) with nucleic acid overlay comparison underneath. The protein is shown as cartoon with EC_T=T_ in blue and 6ASX in grey. The nucleic acids are shown as sticks; EC_T=T_ in yellow with pink T=T, and 6ASX in red.

To understand the possible reasons for stalled EC vulnerability to ribosome-mediated destruction, we determined the structure of the EC stalled at the T=T dimer (EC_T=T_) after incorporation of adenosine monophosphate (AMP) at the first T of the lesion using cryogenic electron microscopy (cryo-EM) (Extended Data Figs. 7 and 8 and Extended Data Table 1). ECs were obtained by walking as above (Methods). We obtained a uniform set of particles, which did not yield more distinct states during classification. Despite an observed orientation bias (Extended Data Fig. 7c), the structure was solved to 2.9 Å resolution (Extended Data Table 1). As expected, the arrangement of nucleic acids in the active site corresponded to the analogous structure of eukaryotic RNAP II stalled after incorporation of AMP at the T=T dimer (post-translocated RNAP II EC_T=T_ in Extended Data Fig. 8c). The overall structure of the stalled EC was similar (r.m.s.d. of roughly 1 Å) to the structures of paused *E. coli* ECs^17,18^ (top of Extended Data Fig. 8d,e). Notably, the β′ clamp of the stalled EC was in the closed (but not swivelled) conformation. The main perturbation we observed was that of the nucleic acids near the active centre of RNAP (where the T=T dimer was located), which translated to surrounding nucleic acids (r.m.s.d. 3.3 and 6.7 Å with two paused ECs; bottom of Extended Data Fig. 8d,e). This shift of nucleic acids suggested a possible weakening of the grip of RNAP on them, which is a major determinant of the stability of an EC in high ionic strength^19^. Indeed, we observed that a stalled EC was much more sensitive to the high salt treatment than a paused EC (Fig. 3b). This structural change may also determine the vulnerability of a stalled EC to ribosome-mediated destruction.

## The Trigger Loop controls EC dislodging

We found that nucleoside monophosphate misincorporation or removal of the 3′-OH group from the 3′ end of mRNA of a paused EC led to significantly increased propensity to dislodging by the ribosome (Fig. 2c and Extended Data Fig. 3e,f). Both ‘wrong’ incorporations are sensed by RNAP catalytic domain, the Trigger Loop^20,21^. We proposed that the vulnerability of the stalled EC to ribosome dismissal may be dictated by the Trigger Loop, which may ‘feel’ the unusual arrangement of nucleic acids. We, therefore, tested the effects of deletion of the Trigger Loop (ΔTL-EC) and of the addition of the antibiotic streptolydigin, that blocks movement of the Trigger Loop^22^, on the sensitivity of the EC to dislodging by the ribosome. The addition of streptolydigin significantly enhanced the stability of both stalled and paused ECs (Fig. 3c). A paused ΔTL-EC was also significantly more resilient to dislodging by the ribosome than the wild-type paused EC (Fig. 3c; we could not test stalled ΔTL-EC because mutant RNAP could not be walked as far as the lesion due to catalytic deficiency). We, however, cannot interpret these findings on the basis of the EC_T=T_ structure, as the Trigger Loop was not resolved in it (Extended Data Fig. 8b,c).

## Interplay with TCR factors, UvrD and Mfd

**Fig. 4:**
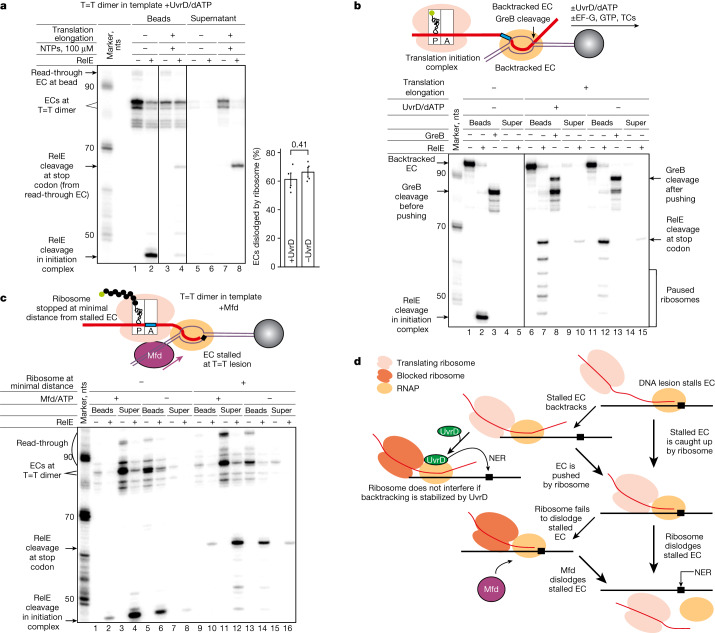
Translation is an alternative pathway to expose DNA lesions during TCR. **a**, Dislodging of the stalled EC by the coupled ribosome is not affected by the presence of UvrD, added before translation elongation. To the right of the gel, the data are presented as mean values ± s.d. from three or more biological replicates (dots); *P* value (two-sided Student’s *t*-test) is shown above to the histogram. **b**, UvrD stabilizes EC in the backtracked state, blocking ribosome-assisted pushing of the backtracked EC. **c**, Ribosomes stopped at a minimal possible distance from the stalled EC do not interfere with the dislodging action of Mfd. **b**,**c**, For gel source data, see Supplementary Fig. 1. Experiments were repeated independently three times with similar results. **d**, A scheme summarizing the proposed model for involvement of coupled translation in TCR and nucleotide excision repair (NER).

Transcription-coupled DNA repair (TCR) requires the stalled EC to be removed from the DNA lesion to make the lesion accessible for repair factors. The helicase UvrD was proposed to expose DNA lesions by causing backtracking of stalled ECs^3^. UvrD, however, will have to act in the opposite direction to the ‘pushing’ ribosome. We, therefore, analysed the possible outcomes of the simultaneous action of both UvrD and a coupled ribosome on an EC stalled at the T=T lesion. As can be seen from Fig. 4a (lanes 3, 7 and quantification), the stalled EC was still dislodged by the translating ribosome in the presence of UvrD. This result means that UvrD may not cause backtracking of the stalled EC or, alternatively, the ribosome may overpower UvrD-mediated backtracking of the stalled EC. To distinguish between these possibilities, we analysed the ability of the ribosome to push an EC that has already undergone backtracking. ECs that elongate to the end of the template and collide with the streptavidin bead undergo stable backtracking^7^, which can be detected using GreB, a factor that cleaves backtracked mRNA in the active centre of RNAP, thus, marking its position (Fig. 4b, lane 3). As can be seen, the ribosome readily pushed these backtracked ECs forward (Fig. 4b, lane 13). However, the addition of UvrD blocked ribosome-mediated pushing of backtracked ECs (Fig. 4b, lane 8). These results indicate that coupled translation could provide a fail-safe mechanism for removal of stalled ECs in situations when an EC has not backtracked from the lesion and when backtracking has happened but was not stabilized by UvrD (scheme in Fig. 4d).

A fraction of the ECs stalled at DNA lesions are not removed by the ribosome, possibly adopting a ‘stable’ conformation. Instead, such ECs stop the coupled ribosome. Another known mechanism for removal of stalled ECs involves the DNA translocase Mfd that can dislodge stalled ECs to expose DNA lesions for repair^2,23^. Mfd acts on the EC at the same interface as a coupled ribosome^24^. We therefore tested whether a ribosome, stopped just behind the stalled EC (thus mimicking a situation when ribosome failed to dismiss a stalled EC), would affect dislodging of this EC from DNA by Mfd. As seen from Fig. 4c (lanes 9, 11), the ribosome did not preclude dislodging of the stalled EC by Mfd. This result indicates yet another fail-safe mechanism for stalled EC removal from the lesion, after ribosome and UvrD-mediated mechanisms have failed (Fig. 4d).

## Discussion

Our results uncover a new translation-dependent mechanism of dislodging of unduly stalled ECs, which functionally and kinetically distinguishes these ECs from paused and active ECs. The functional discrimination by the ribosome between stalled and other ECs is controlled by the Trigger Loop, which may recognize aberrant entities, such as DNA lesions or misincorporated nucleotides, in the RNAP active centre. Although the overall structures of paused and stalled ECs are similar, the local differences in the position of the damaged nucleic acids within the stalled EC is probably responsible for its vulnerability to being dislodged by a coupled ribosome. Notably, however, the stalled EC is resilient to collision with another RNAP trailing on the same DNA, suggesting that the destruction of stalled ECs is specific to collisions with the ribosome.

Kinetic discrimination between active and stalled ECs is based on the ability of active ECs to elongate away from the approaching ribosome, thus minimizing the chance of being dislodged by it. Furthermore, paused ECs are helped into processive elongation by the coupled ribosomes before any of them can be dislodged.

The coupled ribosome begins to pause even before it has reached a paused (but not stalled) EC. In contrast to the blockage of ribosome translocation on collision with a paused EC, this distant pausing is not caused by impaired translocation as the A-site of the paused ribosome remains unoccupied (RelE can bind there). This suggests the existence of as yet unknown crosstalk between two machineries that signals the ribosome to slow down several codons before it reaches the critical distance where EC dislodging may take place. This precautious slowing down of the ribosome may further reduce the probability of dislodging of active ECs. By contrast, such crosstalk does not take place between a ribosome and stalled EC, permitting the ribosome to unobtrusively reach the stalled EC, which, in its turn, is functionally vulnerable to destruction. Sensing of the type of the EC by the ribosome may take place by direct contact between the two machines. However, further structural studies of paused and stalled ECs in the context of the coupled translation will be needed to dissect the mechanistic details of this crosstalk.

Our results indicate that a coupled ribosome can provide a contribution to TCR and nucleotide excision repair by dislodging ECs stalled at DNA lesions (Fig. 4d): (1) the ribosome dislodges stalled ECs (backtracked or not), unless (2) the EC has backtracked and was stabilized in the backtracked state by UvrD and (3) Mfd provides a fail-safe step by dislodging ECs that have failed to be destructed by the ribosome. The ribosome may also contribute to dealing with non-functional ECs that may cause transcription traffic jams^25^ (because trailing RNAPs cannot dislodge stalled ECs), and possibly collisions with other cellular machines such as the replisome. It is tempting to speculate that ribosome-mediated dislodging of faulty ECs may have played an especially important role before the emergence of the dedicated ‘EC-removing’ factors, such as Rho^26^ or Mfd^2^ in bacteria. Eukaryotes, who separated their transcription from translation through compartmentalization, evolved an alternative (but unrelated to bacteria) mechanism for dislodging stalled ECs^27^.

## Methods

### Proteins and nucleic acids

**Extended Data Fig. 9:**
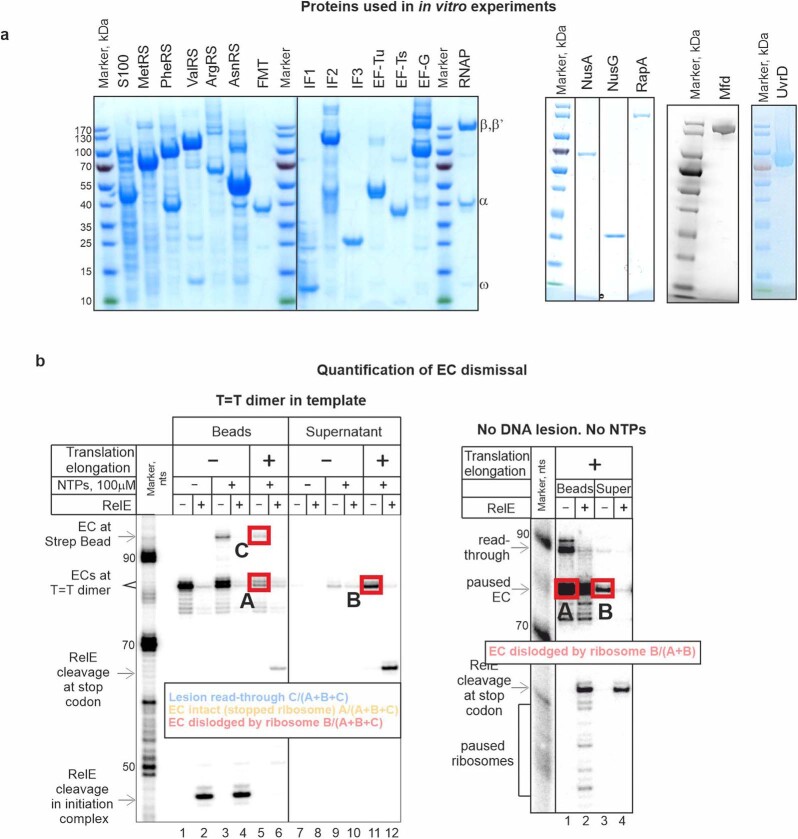
Supporting figures for the Methods section. **a**. SDS gels of purified proteins used in the in vitro coupled system. For gel source data see Supplementary Fig. 1. **b**. Quantitation of the efficiency of dismissal of ECs (on the example of Fig. 2a,d). For the experiments where NTPs were added during translation elongation, the “lesion read-through (C)”, “EC intact (A)” and “EC dislodged (B)” were quantified as a percentage of A + B + C. When NTPs were not added, “EC intact (A)” and “EC dislodged (B)” were quantified as a percentage of A + B.

Wild-type *E. coli* core RNAP was expressed from PVS10 plasmid coding for all five subunits^28^ and purified as described^29^. *E. coli* RNAP lacking the Trigger Loop was from our previous study^20^. The 70S ribosomes, EF-G, EF-Tu, EF-Ts, IF1-3, formyl methionine transferase, methionyl-tRNA synthetase and RelE were purified as described in ref. ^7^. Other individual aminoacyl-tRNA-synthetases were cloned and purified as described for methionyl-tRNA synthetase^7^. Aminoacylation of tRNAs and formylation of Met-tRNA^fMet^ were performed as described^7^, except for the use of individual aminoacyl-tRNA-synthetases in place of S100. Mixtures for translation elongation containing individual ternary complexes were prepared using 80 pmol of aminoacyl-tRNAs, 200 pmol of EF-Tu and EF-Ts, 150 pmol of EF-G, 4 mM GTP in 17 μl of coupling buffer (CB; 25 mM Tris-HCl pH 7.4, 60 mM NH_4_Cl, 10 mM Mg(OAc)_2_, 6 mM β-mercaptoethanol). Mfd^30^, UvrD^31^, RapA^32^, NusG and NusA^7^ were all cloned in pET28a coding for N-terminal 6xHis-tag, purified as described in references, with His-tag subsequently removed by thrombin (Sigma-Aldrich) cleavage as per the suppliers’ instructions. SDS gels of all purified proteins are shown in Extended Data Fig. 9a. Oligonucleotides came from IDT, except for the pyrimidine-dimer (T=T) template from Gene Link. mRNAs were synthesized using T7 RNAP and ^32^P-radiolabelled at the 5′ end as described in ref. ^7^. Oligonucleotides and mRNA sequences are shown in Extended Data Fig. 1.

### EC assembly

The in vitro coupled system for a 20-reaction experiment was assembled as follows: 50 pmol of template DNA and 30 pmol of mRNA were annealed in 22 μl of CB (25 mM Tris-HCl pH 7.4, 60 mM NH_4_Cl, 10 mM Mg(OAc)_2_, 6 mM β-mercaptoethanol), followed by addition of 50 pmol RNAP and then 110 pmol of non-template DNA oligo at 37 °C. ECs were immobilized on 5 µl of streptavidin-Sepharose beads (Cytiva) equilibrated in CB. The system was washed with CB + 1 M KCl and then with CB. The EC was then walked to the desired location on the template with sets of 10 µM NTPs for 3 min per each step (shown in Extended Data Fig. 1 for all ECs) and washing with CB between the steps. For most of the experiments, lesion was reached in one step by addition of CTP (cytidine triphosphate), UTP (uridine triphosphate) and GTP simultaneously. For formation of stably backtracked EC, 1 mM NTPs were added to the EC formed on the template without DNA lesion (Extended Data Fig. 1a) for 5 min. This results in the EC reaching the streptavidin bead, which leads to stable backtracking, as described in ref. ^7^. Then 4 mM GTP was used for misincorporation in place of AMP at the 3′ end of mRNA paused on the template without lesion (Extended Data Fig. 1a). For all reactions, ECs were thoroughly washed with CB and the reaction volume was adjusted to 10 μl. For assessment of translocation state of stalled ECs, 5 pmol of GreA or GreB or 500 µM pyrophosphate (PPi) were added at 37 °C for times indicated in Extended Data Fig. 1b. For the salt stability test, the reaction was transferred to CB + 1 M KCl and left for 10 min at room temperature before separation of supernatant and beads fractions and analysis as described below.

### Coupled transcription–translation

Translation was initiated on the mRNA of the ECs by addition of 20 μl of CB containing 200 pmol of ribosomes, 200 pmol of fMet-tRNA^fMet^, 200 pmol of each of IF1-3 and 4 mM GTP at 37 °C for 8 min. The coupled system was washed with CB, volume adjusted to 25 μl and separated into 5 μl of reactions. Where indicated, the reactions were supplied with 5 pmol of a factor (NusA and NusG, RapA or UvrD) in 3 μl of CB for 2 min at 37 °C. RapA and UvrD reactions also contained 2 mM dATP (final concentration). Translation elongation was started with 17 μl of corresponding elongation mixture of ternary complexes with EF-G/GTP (above). Where indicated, 100 µM NTPs or 400 µM streptolydigin (final concentrations) were added simultaneously with translation elongation mixture. Reactions were allowed to proceed for 4 min at 37 °C. Beads were separated from supernatant. After that, beads were washed with 1 ml of CB and volumes of beads and supernatant fractions were adjusted to 21 μl each. Then 5 µl samples were taken for challenge with 20 pmol of RelE for 5 min at 37 °C or 5 pmol of GreB for 30 s at 37 °C. Reactions were mixed with the equal volume of formamide and EDTA containing buffer. Products were resolved in 10% denaturing (8 M urea) polyacrylamide gel, revealed using phosphorimaging (Cytiva) and analysed using ImageQuant software (Cytiva). The method of quantification of EC dislodging is explained in Extended Data Fig. 9b. Quantitation in figures shows means ± s.d.s from at least three independent experiments. Relevant *P* values are shown above or next to the histograms. Plots were generated using ggplot2 and statistical analyses shown were performed using stat_compare_means (Student’s *t*-test) in RStudio (v.2022.07.2).

### Challenging coupled system with Mfd

For the experiment with Mfd, an EC with longer upstream DNA duplex was used (Extended Data Fig. 1a). The ribosome was allowed to elongate by only F and V codons, thus stopping the ribosome at the minimal distance from the EC stalled at the T=T lesion (25 nts between the active centres of ribosome and RNAP; Extended Data Fig. 1a). The coupled system was washed and volume adjusted as above, and 5 pmol of Mfd and 2 mM ATP were added for 3 min at 37 °C. Beads and supernatant fractions were separated and analysed as above.

### Challenging stalled ECs with trailing EC

Stalled ECs were obtained as above on the nucleic acids scaffold shown in Extended Data Fig. 1e. After washing, 25 pmol of second (trailing) 5′-radiolabelled RNA transcript were added for 5 min at room temperature, followed by addition of 50 pmol RNAP. Complexes were washed with CB and supplied with 20 µM NTPs for 5 min. Supernatant and beads fractions were separated and analysed as above.

### EC preparation for cryo-EM

For cryo-EM, nucleic acids shown in Extended Data Fig. 1a were used, except the non-template strand, which contained a UV-photocleavable group at the biotin end (IDT) allowing for elution from beads. The ECs were prepared in two batches starting with the annealing of 50 pmol of mRNA and 50 pmol of template DNA in 15 µl of CB, followed by the addition of 60 pmol of RNAP for 5 min and 150 pmol of non-template DNA for five further minutes. The ECs were immobilized on 12 µl of streptavidin bead slurry and washed with CB + 1 M KCl and then CB. The ECs were walked to the T=T lesion using 20 µM GTP, CTP and UTP (final concentration) followed by CB washes. 20 µM ATP was added for 3 h. Volumes of reactions were adjusted with CB to 50 µl. Stalled ECs were eluted from the beads by exposure to 365 nm light of the lamp BDH VL-206BL (Vilber-Lourmat) equipped with T-6L light tubes for four rounds of 30 s. Supernatants were joined and concentrated to 25 µl on Amicon-50 0.5 ml filter (Merck Millipore).

### Cryo-EM grid preparation

UltrAuFoil300 R1.2/1.3 holey gold grids (Quantifoil) were positively glow-charged using an EasyGlow Discharge System (PELCO) at 25 mA for 4 min at 0.26 mBar. This was followed by three applications of 3.5 µl of eluted ECs using a Vitrobot Mark IV (FEI) with 100% chamber humidity at 4 °C, before plunge-freezing into liquid ethane.

### Cryo-EM data acquisition and processing

The workflow and statistics of cryo-EM analysis are shown in Extended Data Fig. 7a and Extended Data Table 1, respectively. Grids were imaged using a Glacios cryo-TEM (Thermo Scientific), with a Falcon 4 electron detector (Thermo Scientific), at the York Biostructure Laboratory (York University). A total of 16,264 videos were recorded in EPU (Thermo Scientific) with a nominal magnification of ×240,000 and pixel size of 0.574 Å/pix with a defocus range of −0.8 to −2.0 µM. Data were collected with a 6.4 s exposure, 1,574 subframes (total frames) and dose per frame of 0.03246 electrons per Å^2^ to give a total dose of 50 electrons per Å^2^.

Videos were motion corrected using motioncorr2 (ref. ^33^) before estimation of the contrast transfer function (CTF) with CTFFIND4 (ref. ^34^), in RELION^35^. These videos were also motion corrected and had CTF estimated using cryoSPARC implementations^36^ to allow use of iterative cryoSPARC two-dimensional (2D) particle sorting algorithms starting with an initial blob pick using a 100–300 pixel box range and 15 Å lowpass filtered micrograph images, finding 2,431,144 particles. The final 160,183 particles were transferred from cryoSPARC and extracted in RELION with a box size of 500 pixels, 5 × 5 binned to 100 pixels with a pixel size of 2.87 Å/pix and subject to several rounds of further 2D classification and particle selection. The 143,018 particles from the final selection of 2D classification were then used to generate an initial model through a RELION gradient-driven algorithm with a 250 Å mask diameter. This initial model was converted and upscaled using the RELION command line image handler to a box size of 500 pixels with a pixel size of 0.574 Å/pix, while the particles were re-extracted with the same box and pixel sizes. The particles were then subjected to three-dimensional (3D) classification enabling the further removal of junk particles, leaving 131,098 particles for 3D auto refinement. The refined map was then postprocessed to 3.1 Å before advanced particle processing using CTF refinement and Bayesian polish jobs. The subsequent final 3D refinement and postprocessing was carried out with a soft mask of the entire EC, resulting in a final map resolution of 2.87 Å, as reported by RELION.

### Model building and refinement

An initial model was rigid fit to the final map in ChimeraX (UCSF) using the cryo-EM data-generated model of the *E. coli* RNAP EC^37^ (Protein Data Bank (PDB) ID 8FVR). This model was then subjected to real space refinement in phenix^38^ and manual editing in COOT^39^. The T=T lesion was built in place of template DNA residues as a ligand using a T=T lesion from a T7 RNAP stalled at the T=T lesion^40^ (PDB ID 1SL2). Extra DNA and RNA extensions to the main chains were completed in Coot, before further cycles of refinement and processing in phenix and Coot. Point spread function resolution (Extended Data Fig. 7c) was calculated using cryoEF^41^.

### Reporting summary

Further information on research design is available in the Nature Portfolio Reporting Summary linked to this article.

## Online content

Any methods, additional references, Nature Portfolio reporting summaries, source data, extended data, supplementary information, acknowledgements, peer review information; details of author contributions and competing interests; and statements of data and code availability are available at 10.1038/s41586-023-07014-3.

### Supplementary information

Supplementary Figure 1 Uncropped gels.

Reporting Summary

## Figures and Tables

**Extended Data Table 1:**
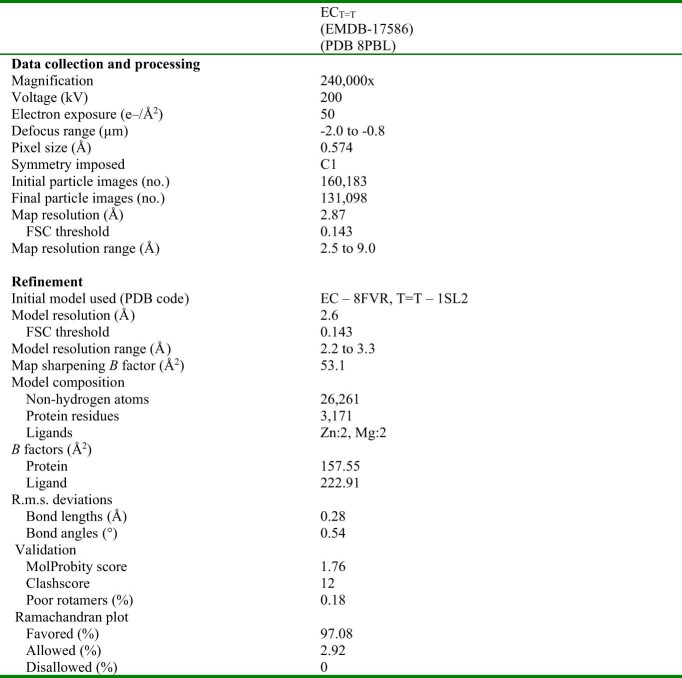
Cryo-EM data collection, refinement and validation statistics

## Data Availability

The final coordinates were deposited to PDB with the accession code 8PBL, and the cryo-EM map was deposited to the Electron Microscopy Data Bank, under the code EMD-17586. Plasmids are available upon request.
