# Supplementary material for: Translation selectively destroys non-functional transcription complexes
Source: Nature. 2024 Feb 7;626(8000):891–6. doi: 10.1038/s41586-023-07014-3 (PMC10881389; doi:10.1038/s41586-023-07014-3)
Supplement: Supplementary file 1 — Uncropped gels. [file 41586_2023_7014_MOESM1_ESM.pdf]

nature portfolio

https://doi.org/10.1038/s41586-023-07014-3

Supplementary information

Translation selectively destroys non-functional transcription complexes

In the format provided by the authors and unedited

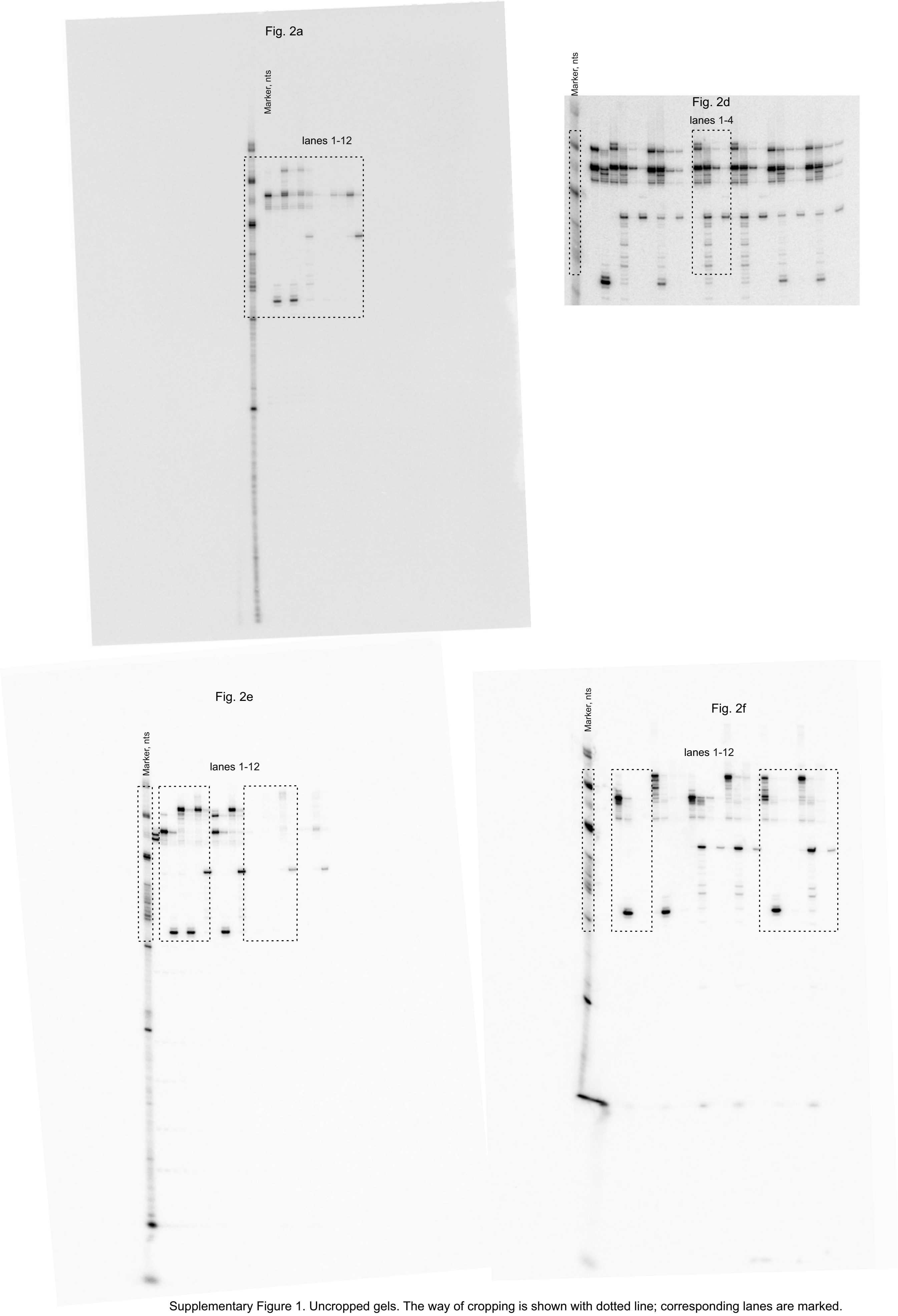

Supplementary Figure 1. Uncropped gels. The way of cropping is shown with dotted line; corresponding lanes are marked.

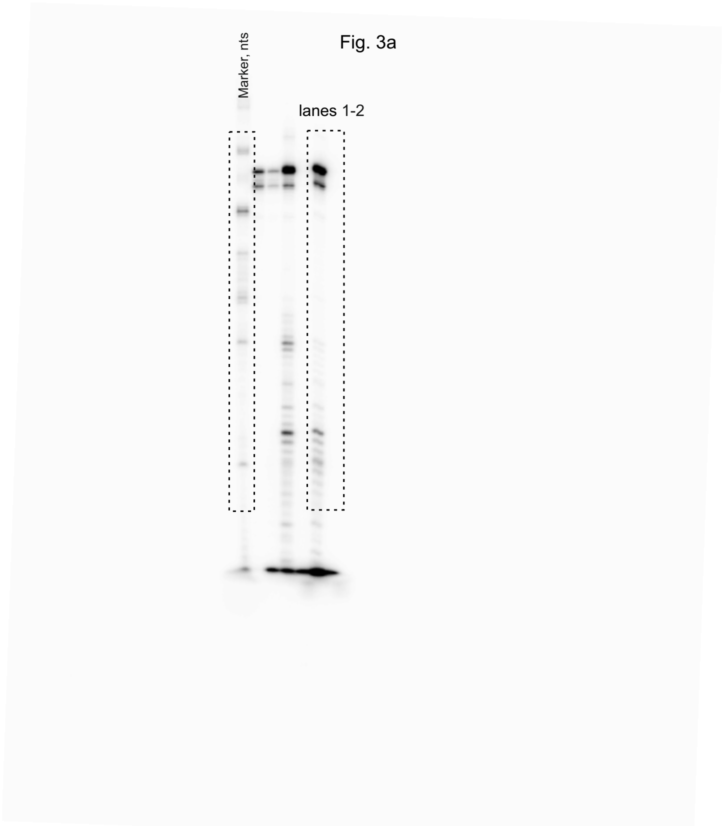

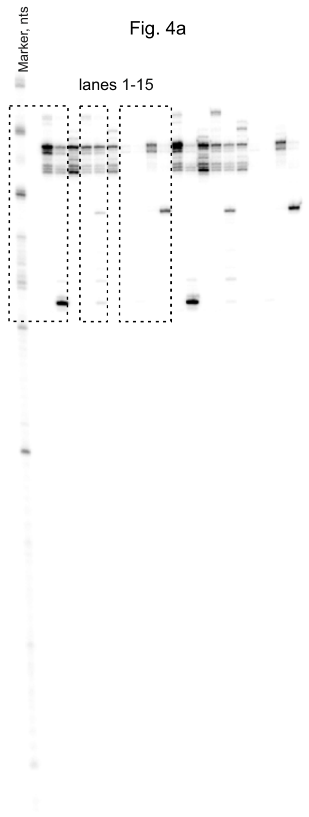

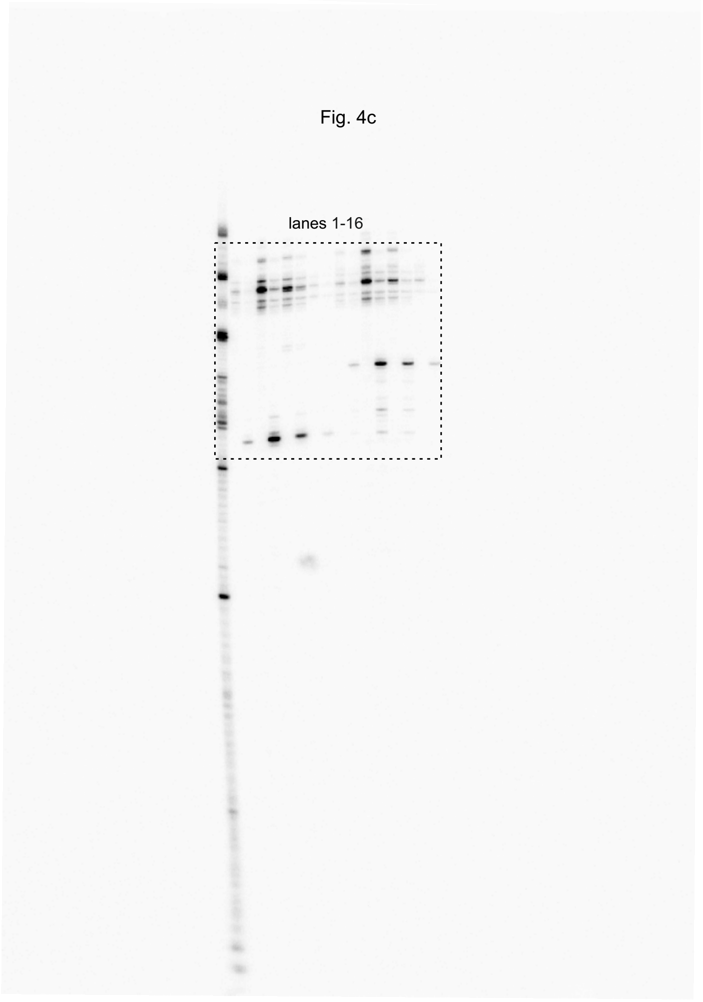

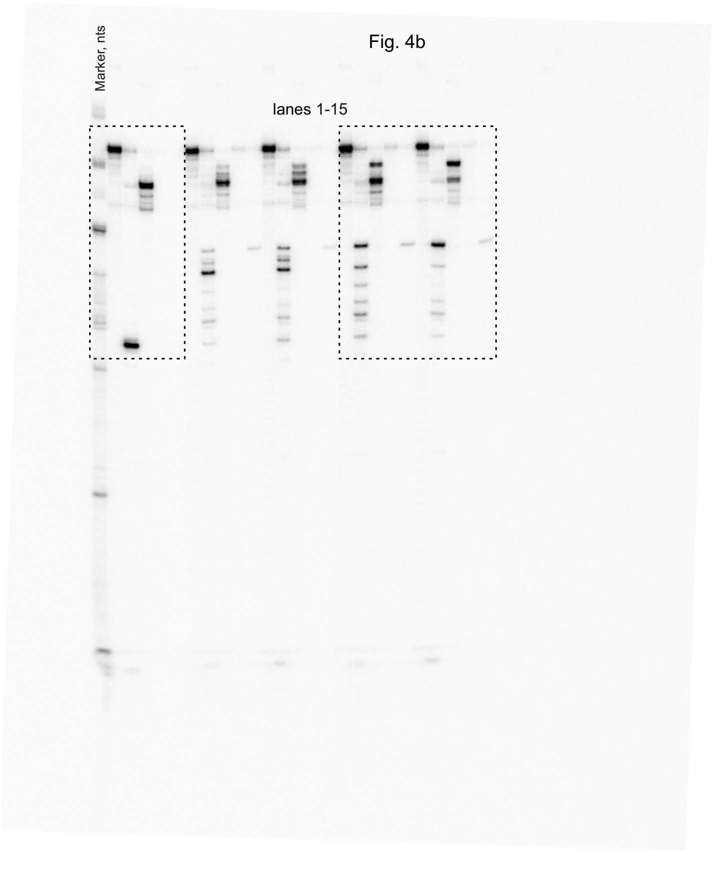

Supplementary Figure 1 (*continued*). Uncropped gels. The way of cropping is shown with dotted line; corresponding lanes are marked.

Extended Data Fig. 1b

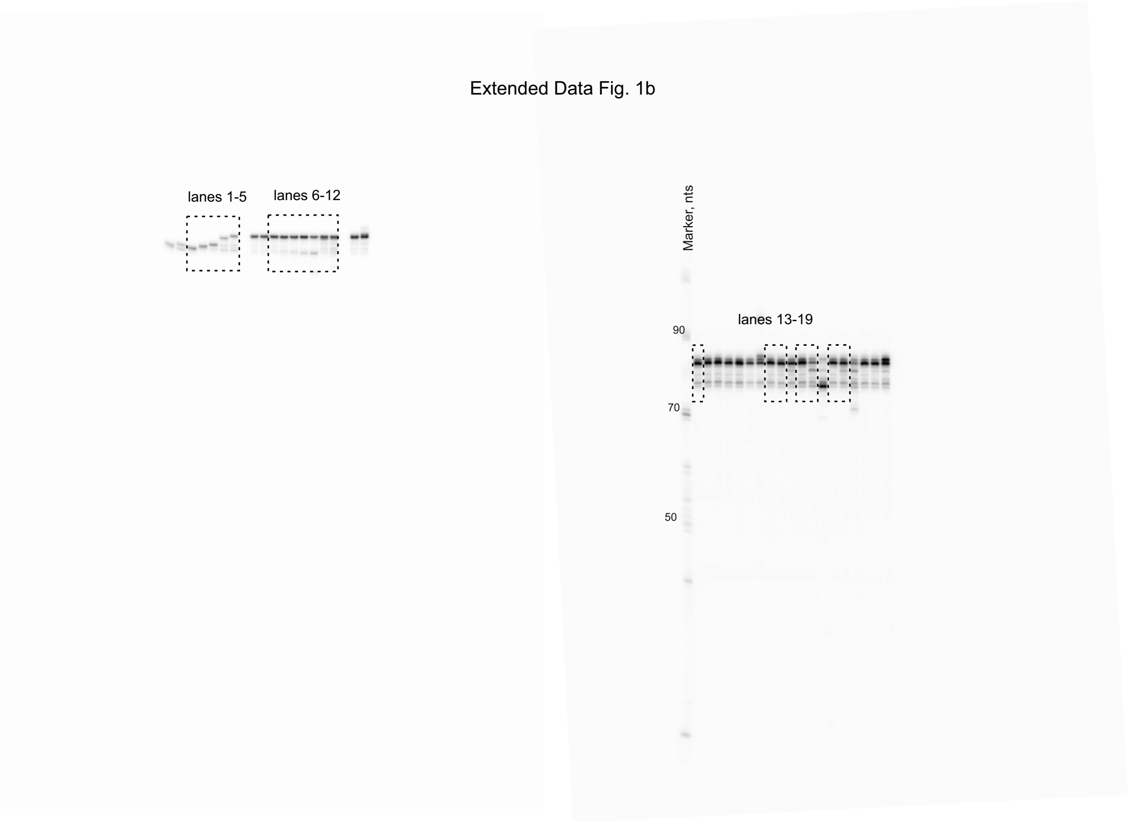

Extended Data Fig. 3b

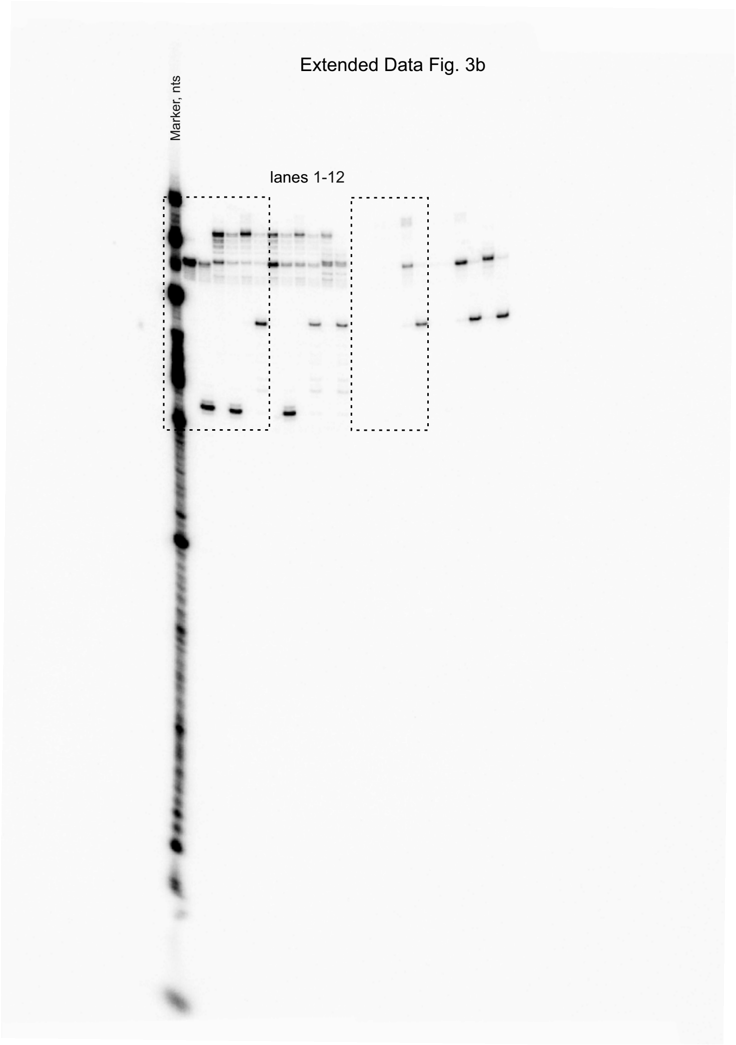

Extended Data Fig. 3c

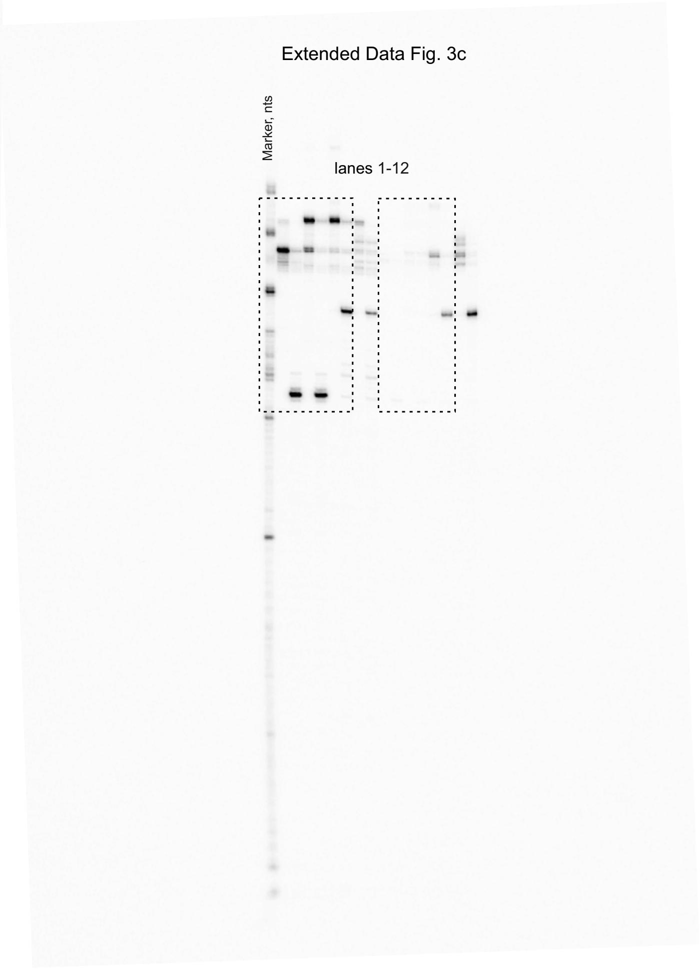

Supplementary Figure 1 (*continued*). Uncropped gels. The way of cropping is shown with dotted line; corresponding lanes are marked.

Extended Data Fig. 3d

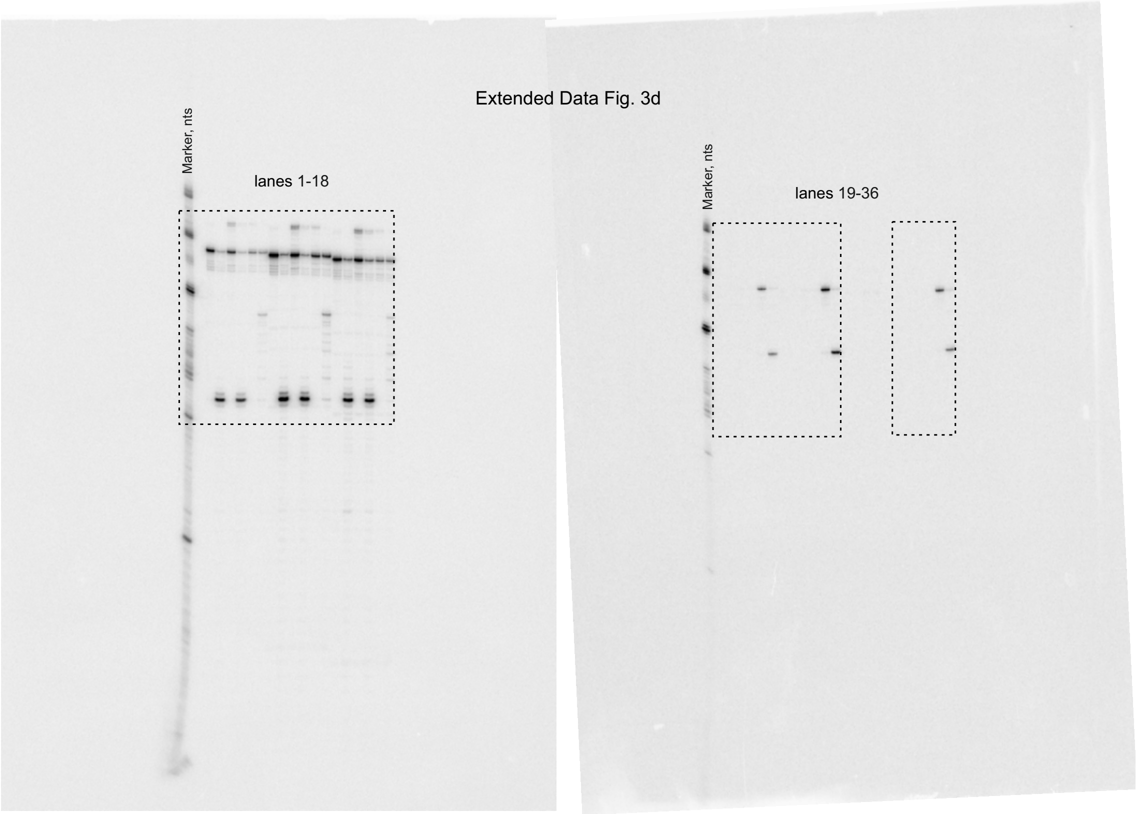

Extended Data Fig. 3e

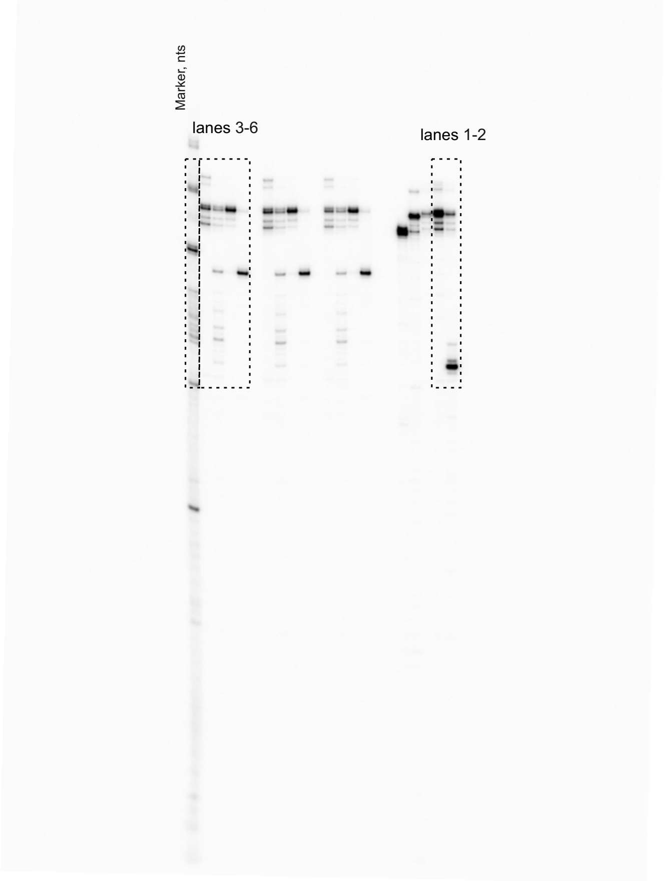

Extended Data Fig. 3f

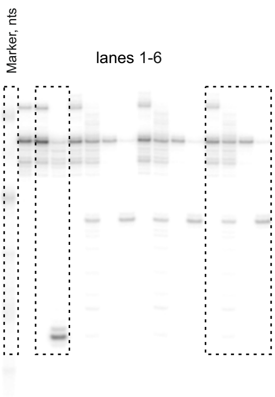

Supplementary Figure 1 (*continued*). Uncropped gels. The way of cropping is shown with dotted line; corresponding lanes are marked.

Extended Data Fig. 4a

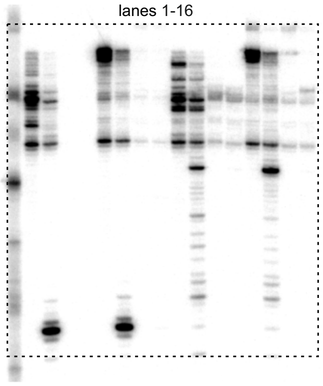

Extended Data Fig. 4b

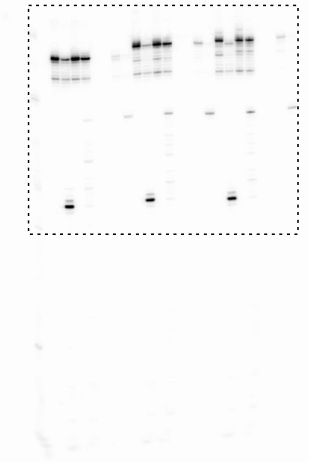

Extended Data Fig. 6

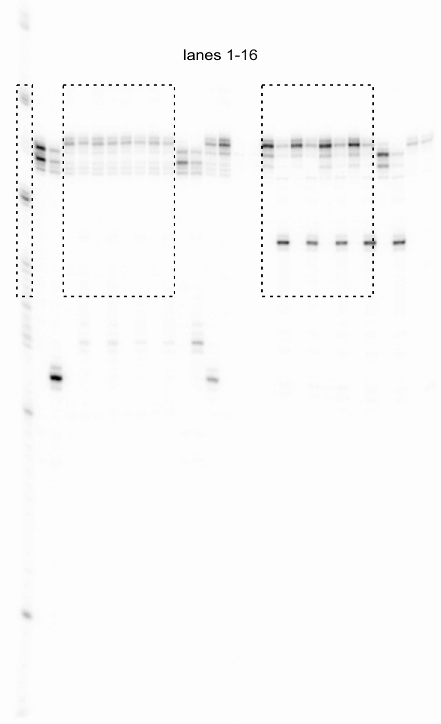

Supplementary Figure 1 (*continued*). Uncropped gels. The way of cropping is shown with dotted line; corresponding lanes are marked.

Extended Data Fig. 9a

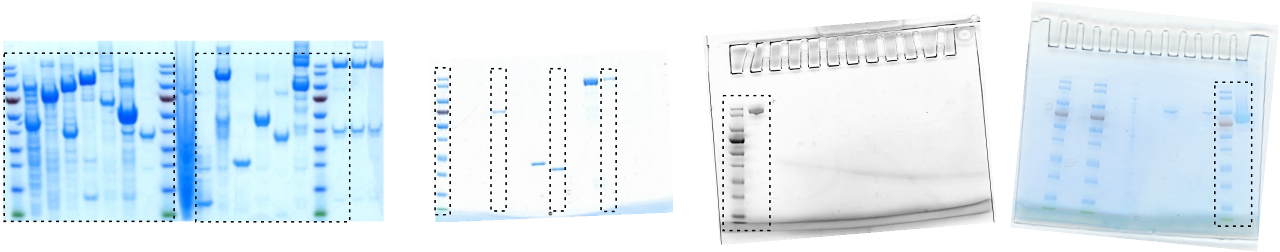

Supplementary Figure 1 (*continued*). Uncropped gels. The way of cropping is shown with dotted line; corresponding lanes are marked.
